# Viral and bacterial co-infection in pneumonia: do we know enough to improve clinical care?

**DOI:** 10.1186/s13054-016-1592-y

**Published:** 2017-01-26

**Authors:** Kelly A. Cawcutt, Andre C. Kalil

**Affiliations:** 1 Department of Internal Medicine, Division of Infectious Diseases, University of Nebraska Medical Center, Omaha, NE USA; 2 University of Nebraska Medical Center, 985400 Nebraska Medical Center, Omaha, NE 68198 USA

**Keywords:** Viral, Bacterial, Pneumonia

Both bacterial and viral pneumonia are well accepted entities; however, with evolving diagnostics there has been increasing interest in the pathogenesis, epidemiology, presentation, and prognosis of viral pneumonias. In addition, the concept of viral and bacterial co-infections in pneumonia is an area of growing research and may be best recognized among patients with influenza who develop secondary bacterial infections; there was particular interest in this after the 2009 pandemic [1–4]. However, in the setting of increasing molecular diagnostics, particularly multiplex PCR platforms, there is an opportunity to better define the epidemiology of co-infections and their impact on clinical diagnosis and patient outcomes [5, 6]. There is some evidence that dual infection may worsen patient outcome, including severity of disease and mortality [1, 2]. With this in mind, the article by Voiriot et al. [7], “Viral–bacterial coinfection affects the presentation and alters the prognosis of severe community-acquired pneumonia”, adds to our expanding knowledge base in this arena.

Prior studies have focused on community-acquired pneumonia (CAP) with multiplex pathogen panels, and rates of positivity for concurrent viral detection have ranged up to approximately half of patients; however, detecting a virus does not necessarily imply the virus is directly affecting the patient’s current clinical illness [1, 3, 4, 8]. The study by Voiriot et al. is a single center, retrospective study of critically ill patients admitted to an ICU between October 2011 and June 2015 for community-acquired pneumonia who also had a multiplex retrograde pathogen panel completed within 72 h of admission to the ICU. Patients were categorized based on pathogen, including bacterial, viral, mixed viral and bacterial pathogens, and no etiology based on microbiologic data obtained. The primary endpoint was to identify specific features regarding presentation and prognosis of mixed viral bacterial co-infected patients. Clinical course was evaluated via a composite criterion of “complicated course” including hospital death or utilization of mechanical ventilation for greater than 7 days. The study included 174 patients that were predominantly male and of old age. Patients with microbiologic documentation of infection included 144 patients: 52.3% had at least one bacterium identified and 56.3% had at least one virus identified. Of these, 45 had a mixed bacterial and viral pneumonia per the investigators’ criteria; 15 were influenza and 14 were picornavirdae. In the analyses, the composite “complicated course” achieved statistical significance, with higher rates in the mixed group. Presentations were also altered, with increased groundglass opacities radiographically and increased severity of illness among those with mixed infection. It is of note, however, that radiographic findings for bacterial and viral pneumonia may have significant overlap and have historically not been particularly predictive of single entity, dual, or mixed infection [1].

This retrospective study has several potential limitations (e.g., selection bias, confounding by indication of ordering or not the multiplex PCR panel) but the results, limited as they may be, demonstrate that over half of patients admitted to an ICU with presumed community-acquired pneumonia have detectable virus at the time of presentation, consistent with other studies noted above. In the study by Voiriot et al., dual infection with mixed bacterial and viral pathogens was associated with worsening patient outcomes. Interpretation of the study is somewhat limited as it is difficult to ascertain the clinical relevance of the detection of virus given the underlying knowledge that viral shedding is not uncommon. Viral shedding could be a surrogate marker for disease severity associated with the primary bacterial pneumonia. However, the worsened composite outcome would suggest that, regardless of colonization, shedding, or active infection, the presence of concurrent virus may hold prognostic value. From a clinician standpoint this becomes increasingly of concern as we detect episodes of mixed infection as very few of the viruses detected on multiplex platforms have active treatment options. Further, it can be difficult in particular patients, such as those with underlying pulmonary disease, to ascertain what may be bacterial colonization or shedding versus active viral infection [6, 8]. The study by Voiriot et al. did not include discussion of prior infections, particularly in patients with an existing immunocompromised state that may have predisposed them to persistent viral shedding, which can last for months.

With all of this in mind, the study by Voiriot et al. reinforces prior literature that CAP may be far more complex than has been historically recognized, with potential dual active infections, possible immune-modulating effects of some viruses, interactions of the pulmonary microbiome, and variations in clinical presentation and prognosis that may be secondary to direct pathogen effect versus host immune response, or the combination of all of the above [1, 8]. Further prospective studies are needed to better determine the role of these viruses and their interactions in patients with CAP. Unfortunately, at this time the knowledge of dual infection does not guarantee changes in clinical practice that would impact individual patient outcomes given the lack of proven causal effects from viral–bacterial co-infections, as well as the paucity of effective antiviral therapy available. Detecting a single virus as the pathogen would ideally equate to improved antimicrobial stewardship, but the legitimate concern for dual infection may preclude this [5]. However, it is of great importance that recognition of active viral replication may portend clinical value from the infection prevention standpoint as appropriate isolation may prevent nosocomial spread of viral illnesses, particularly given the high rate of virus detection [5, 9]. Finally, preventative strategies with vaccination for both bacterial and viral pathogens remain of paramount importance.

## Abbreviations

CAP: community-acquired pneumonia
PCR: polymerase chain reaction
